# Exploiting hot-spots; effective determination of lichen diversity in a Carpathian virgin forest

**DOI:** 10.1371/journal.pone.0203540

**Published:** 2018-09-13

**Authors:** Jan Vondrák, Jiří Malíček, Zdeněk Palice, František Bouda, Franz Berger, Neil Sanderson, Andy Acton, Václav Pouska, Roman Kish

**Affiliations:** 1 Institute of Botany of the Czech Academy of Sciences, Průhonice, Czech Republic; 2 Department of Botany, Faculty of Science, University of South Bohemia, České Budějovice, Czech Republic; 3 Charles University in Prague, Faculty of Sciences, Department of Botany, Czech Republic; 4 National Museum, Department of Mycology, Cirkusová, Horní Počernice Czech Republic; 5 Independent Researcher, Kopfing, Austria; 6 Independent Researcher, Southampton, United Kingdom; 7 Independent Researcher, Taynuilt, United Kingdom; 8 Faculty of Forestry and Wood Sciences, Czech University of Life Sciences Prague, Kamýcká, Suchdol, Czech Republic; 9 Laboratory for Environmental Protection, Uzhhorod National University, Uzhhorod, Ukraine; Fred Hutchinson Cancer Research Center, UNITED STATES

## Abstract

Although lichenized fungi are among the most reliable indicators of forest quality and represent a considerable part of forest biodiversity, methods maximizing completeness of their species lists per area are lacking. Employing a novel methodological approach including a multi-expert competition and a search for local hot-spot plots, we have obtained outstanding data about epiphytic lichen biota in a part of the largest Central European virgin forest reserve Uholka–Shyrokyi Luh situated in Ukrainian Carpathians. Our field research consisted of two four-day periods: (1) an overall floristic survey and a search for spots with raised lichen diversity, and (2) survey in four one-hectare plots established in lichen diversity hot-spots along an altitudinal gradient. Recorded alpha-diversities in plots ranged from 181–228 species, but estimated species richness is in the range 207–322 species. Detected gamma-diversity was 387 species; estimates are 409–484 species. 93% of the species found in the forest were recorded in plots, but only 65% outside the plots. This underlines the high-efficiency of the multi-expert competitive survey in diversity hot-spot plots. Species richness in each one-hectare plot was equal to the numbers of species obtained by floristic surveys of much larger old-growth forest areas in Central Europe. Gamma-diversity detected in the Uholka primeval forest far exceeded all numbers achieved in Central European old-growth forests. Our method appears to be both effective (it obtains a more nearly complete inventory of species) and practical (the resources required are not unreasonably large).

## Introduction

Forests have the highest biodiversity among terrestrial biomes [1]. Regrettably, many natural forests have been destroyed during the last few centuries. In some regions, including Central Europe, pristine forests have almost vanished, being altered by land use, and only fragments remain. These remnants support the greatest diversity of many forest organisms, among them epiphytic and epixylic lichens (e.g. [2–6]) which are considered the most reliable indicators of forest-continuity and forest quality [7,8]. Tiny crustose lichens, that are often neglected, are especially sensitive to environmental changes as they are intimately associated with microhabitats [9,10].

It has been shown many times that cryptogam diversity in old-growth forests is not uniformly distributed (e.g. [11–13]), and that large parts of these forests (much more than 50%) have rather low local diversity. Species richness of epiphytic lichens is much greater in hot-spots, such as humid valley bottoms, ridges with rock outcrops, gaps and screes or timber-line forest edges [14,15]. Furthermore, the diversity is not equally distributed within particular forest habitats. Each habitat has a specific variability in microhabitats and substrates suitable for numerous niche-specific lichens [16–18]. In old-growth forests, lichen diversity is positively correlated with the amount of variety in the forest structure [19–22], which is influenced by microclimatic and soil conditions and by natural disturbances. Diversity of lichens is not uniformly distributed in the vertical dimension either. Species composition and trait diversity of lichens in canopies differ strongly from that on tree trunks [23,24].

The simplest measure of the quality of a forest is its total biodiversity, i.e. the total number of species present. Although conceptually simple, actually obtaining it is far from simple: it is challenging, even for experts, to determine all (or nearly all) the species present. Remnants of several important European old-growth forests have been surveyed for lichen diversity using a variety of methods, including taxonomic surveys and ecological sampling [25], most of which were based on random records [15,26,27]. However, in a very heterogeneous environment random sampling can not obtain an inventory that is near to complete (unless the area under study is tiny, or the survey is impractically large). It will tend to omit specific microhabitats and locally rare species. Unrelated to that problem, there is the further difficulty that some lichen species are small, inconspicuous and very easily overlooked.

A related problem is that of plot size. If individual plots are large, the problems discussed above for the forest as a whole will occur (though to lesser degree) for each plot. That will result in incomplete and/or biased species lists for each plot, which makes comparisons between plots difficult or meaningless. The obvious solution is to use numerous small plots (much less than 1 hectare) that can be surveyed more easily [3,13,28–35]. This does facilitate some kinds of statistical data processing [25], but it creates new problems: (1) Rare species (which may have significant bioindicative value) are unlikely to be sampled [12]; (2) Uncommon substrates and microhabitats (which may have numerous species with specialised requirements) are unlikely to be sampled; (3) The majority of the plots will be in "boring" parts of the forest with low biodiversity and the rare localities with high biodiversity ("hot-spots") are unlikely to be sampled adequately, or at all. Although these problems can be reduced by increasing the number of plots, they can not usually be reduced enough unless the number of plots is increased to a level that would require impractical resources of time, manpower and money if each plot is to be surveyed thoroughly [36]. In practice, use of a large number of plots is likely to mean that plots are not surveyed thoroughly.

These kinds of problems led some researchers to try a different method: a detailed diversity survey in larger plots (one to several hectares), but with no or few repeats within a locality [37–39].These surveys were always performed by more than one researcher, which results in better recording of rare and inconspicuous species, as each researcher has different skills. Although this method can yield almost complete species lists per plots, it requires an enormous sampling effort. For example, Lõhmus et al. [38] reported an astonishing 500 person-hours for a single 2-hectare plot. This usually makes repeated surveys impractical. We are inclined to employ larger plots (we chose 1 hectare squares), but we realized that their placement in the forest must be carefully selected to avoid impractically large sampling effort.

Lichenologists do not seem to have available a sampling method that (1) allows meaningful comparison between different localities, (2) requires only a feasible amount of resources, and (3) yields species lists that are reasonably close to complete. As regards the first point, we extracted lichen diversity data from numerous surveys of Central European forests [39,40], and we concluded that the species lists are hardly comparable. They are strongly affected by the different survey methods used and the different skill sets of those who did the surveys. Here we propose a method that, in our view, goes a long way towards meeting all three goals.

Our method combines the multi-expert competitive approach [39] with a search for local diversity hot-spots. The former ensures that any plot that is studied will be studied very thoroughly, and the resulting species list for it will be close to complete. The latter ensures that all (or at least most) biologically important aspects of the forest will receive such attention. Together, these ensure that goal (3) above will be met. The resource requirements, though obviously greater than if only a single worker were to make the survey, are not excessive: goal (2). The method can be applied in a standardised way, which should ensure that goal (1) is met.

Our aim in the work reported here was simply to use the method to survey epiphytic lichens in a single large forest and to determine whether it worked, i.e. to determine whether we had overlooked any serious problems. Basically, that meant demonstrating that it meets goals (2) and (3). Our aim was not to confirm formally that it meets goal (1), though we expect that it does. Formal confirmation of that will require surveys of several forests and is beyond the scope of the present paper which is based on a research in the largest Central European primeval forest, Uholka–Shyrokyi Luh in the Ukrainian Carpathians.

## Methods

Administration of the Carpathian Biosphere Reserve (Rakhiv, Ukraine) provided permission for our research.

### Surveyed area and timing

**Fig 1:**
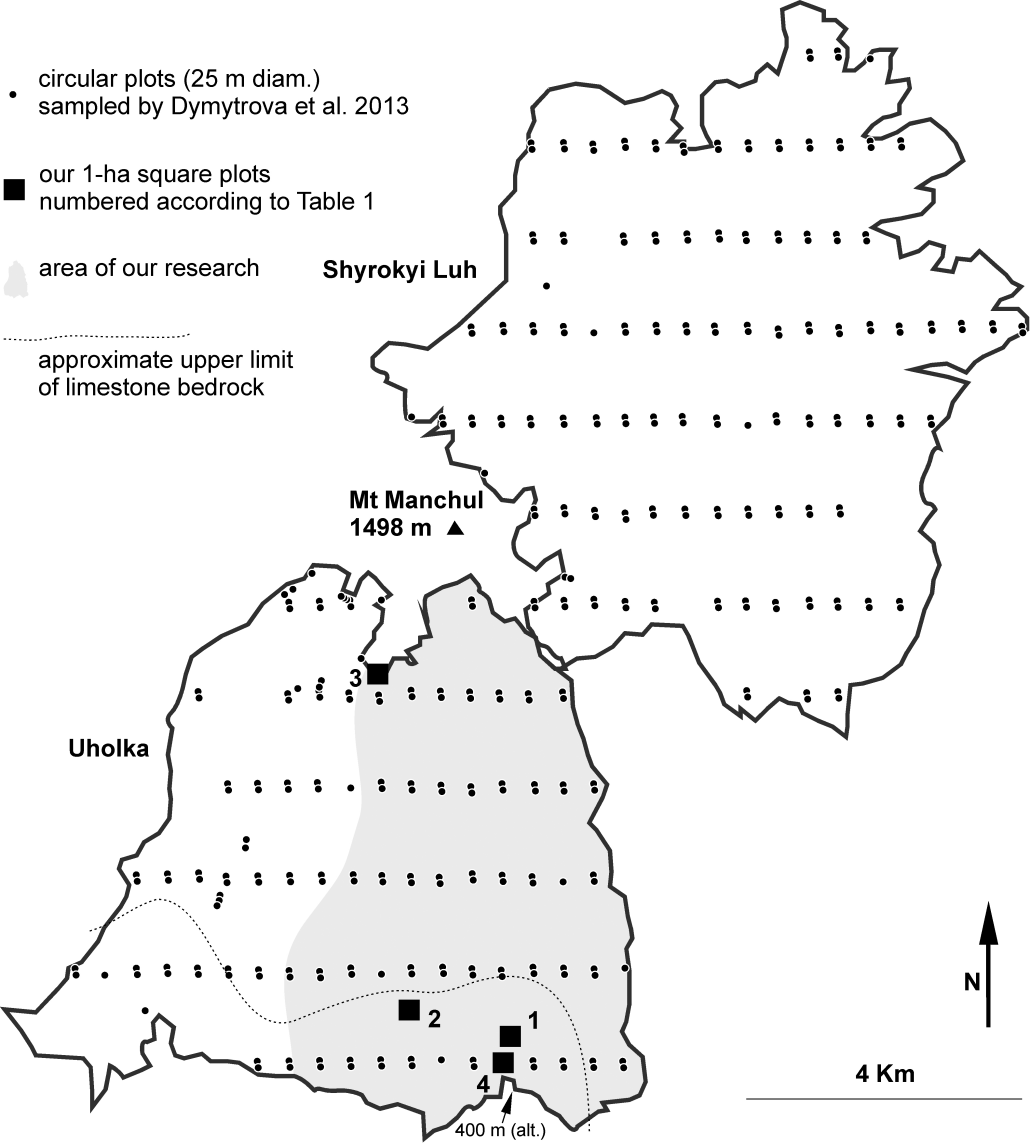
Sampling area. Protected area of old-growth beech forest “Uholka–Shyrokyi Luh” surveyed by Dymytrova et al. [33] by a systematic sampling on circular plots of 500 m^2^ (black dots). The area is divided into a southern part, Uholka, and the northern one, Shyrokyi Luh. We surveyed only a part of Uholka, the valley of the brook Velyka Uholka (area in grey) where we selected four plots (black squares) in hot-spots of lichen diversity. Forest habitat diversity is distinctly greater at lower altitude, in the area with limestone bedrock (below the dotted line).

We surveyed one of the Carpathian Biosphere Reserves, “Uholka–Shyrokyi Luh” (c. 30 km NE of Khust, western Ukraine), one of the largest old-growth forest complexes in Europe with 10400 ha [41]. It was systematically surveyed for lichen diversity by Dymytrova et al. [33], so we can compare our results with theirs. Rare lichen species known from the locality [33] indicate that the forest is rich in lichen species. We surveyed a 2300 ha part called Uholka, on the southern slopes of Mt Menchul (Fig 1). The terrain is rugged, often formed by steep slopes, separated by numerous valleys with watercourses at altitudes 400–1200 m. The area is dominated by *Fagus sylvatica*, but the forest is not homogeneous throughout (see [41] for details). Our field research in May 2015 lasted eight days; four days for seeking suitable plots and conducting an overall lichen diversity survey, four more for surveys in plots (see below).

### Stratified non-random plot selection

**Fig 2:**
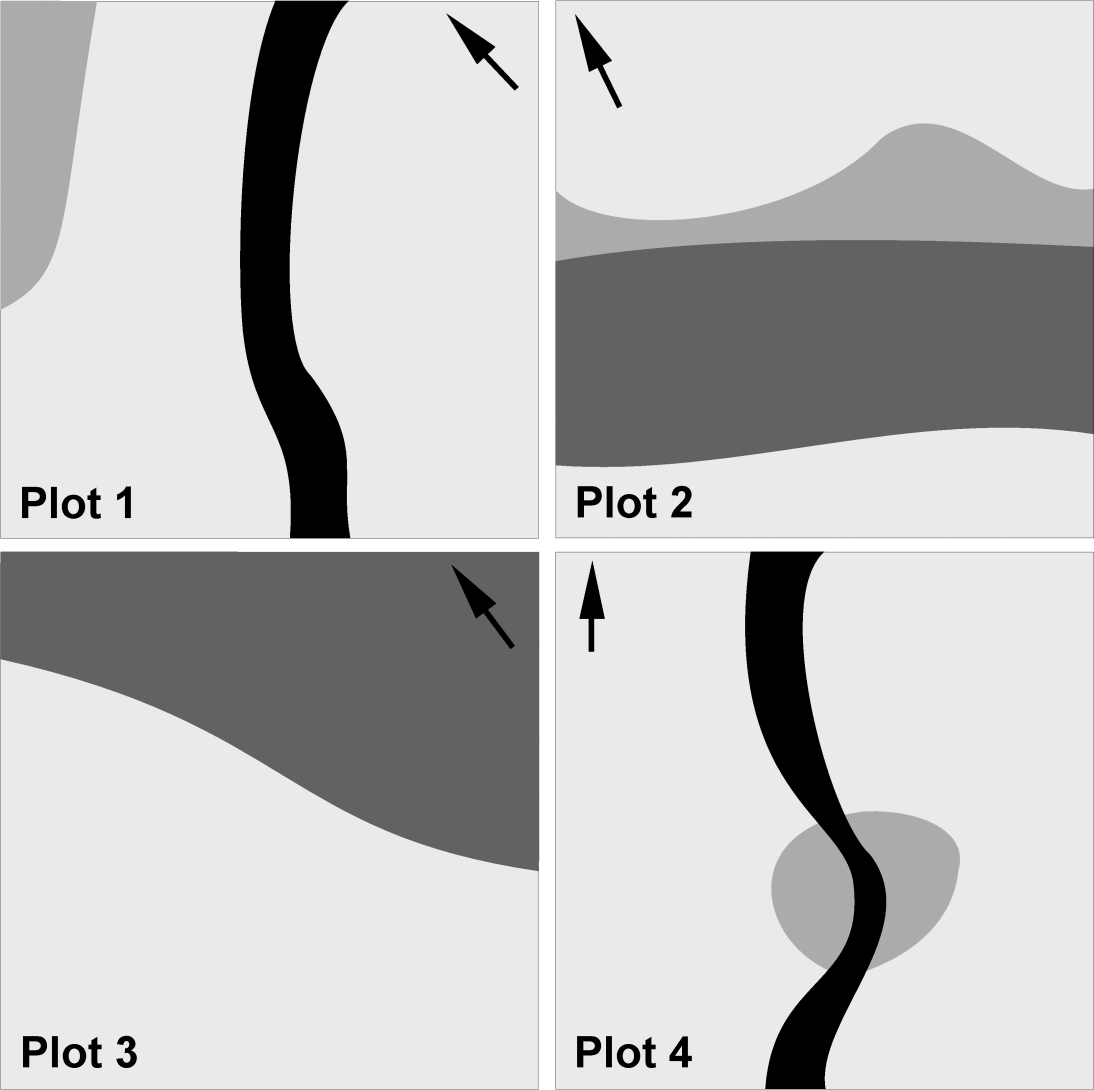
Variability of forest habitats in surveyed plots. The prevailing forest type, a dense beech forest without intermixed tree species, is present in all plots (pale grey). Wet ravine forest with common *Carpinus betulus* is present in the lowermost plots 1 and 4 (black). Sun-lit mixed forest on limestone rocks and scree (medium grey) is present in plots 1, 2 and 4. Damp mixed forest on steep slope with limestone outcrops, dominated by *Acer platanoides*, *A*. *pseudoplatanus*, *Fraxinus* and *Tilia*, is present in plot 2 (dark grey). Sparse beech forest occurs in plot 3 (dark grey) at the artificially lowered timber line with the occurrence of large, old and deformed trees with weathered bark. Lower parts (up to 2 m height) of beech trunks in this forest type are sun-lit due to summer grazing and often harbour more than 40 lichen species.

The first four days were devoted to a search for hot-spots. We wished to find four 1 ha plots that could be expected to include most of the lichen biodiversity present in the forest. Our own field experience and discussions in the literature [12,13,24] indicated that we should look particularly for: (1) a multilayered canopy indicating a non-even-aged forest; (2) the presence of over-mature, dying and dead trees with weathered and mossy bark; (3) the presence of both standing and lying dead wood; (4) the highest diversity of tree species at the local scale; (5) the presence of small natural forest gaps; (6) the availability of canopy lichens on fresh windthrows or at least on fallen big branches (as we had no other way to survey canopy lichens). These criteria are usually met in sites where several different forest habitats meet and where the length of ecotones is maximized. We established two plots at low altitude in a deep valley, one at medium-altitude on a limestone ridge and one at the upper forest limit (Fig 1); their midpoints were localized by GPS (Table 1). The four plots contained most of the forest habitat types present in the area (Fig 2). The predominant forest type, a dense beech forest without any other intermixed tree species, covering more than 99% of the studied area, was included in all plots.

**Table 1:** Surveyed one-hectare plots in the Uholka forest.

	Coordinates	mean alt. (m)	available substrates (rare, in brackets)	research intensity
Plot 1	48.250831N, 23.696454E	510	FS, CB, logs, snags, (AP, Apl, CA, FE, SN, UG)	7 researchers / 6 hours
Plot 2	48.256089N, 23.661366E	800	FS, AP, Apl, CA, CB, FE, TB, TIL, UG, logs, snags, (QU, SA)	6 researchers / 6 hours
Plot 3	48.297948N, 23.666583E	1200	FS, logs, snags	7 researchers / 6 hours
Plot 4	48.244879N, 23.694648E	430	FS, CA, CB, logs, snags, (AP, Apl, FE, SN, TIL, UG)	7 researchers / 6 hours

Substrate abbreviations: Apl, *Acer platanoides*; AP, *Acer pseudoplatanus*; CA, *Corylus avellana*; CB, *Carpinus betulus*; FE, *Fraxinus excelsior*; FS, *Fagus sylvatica*; QU, *Quercus*; SA, *Sorbus aucuparia*; SN, *Sambucus nigra*; TIL, *Tilia*; UG, *Ulmus glabra*. Substrates in brackets are not common in plots.

The "recipe" for locating a hot spot is thus: seek a site that has several of the six factors listed above (the more the better). Each of those factors is easy to spot visually, because it corresponds to something that is different from "the bulk of the forest", so our method is not difficult to apply. A future worker would have no difficulty locating hot spots, though they would probably be different hot spots than ours.

### Multi-expert competitive survey

In the second stage, each of the four selected plots was surveyed by a team of experienced lichenologists (the first seven authors) using the competitive method [39]. The surveys were conducted by 7 experienced lichenologists over a period of 4 days. That may sound like a lot of manpower, but Uholka–Shyrokyi Luh is a very large forest. Smaller forests could be surveyed with fewer resources.

**Fig 3:**
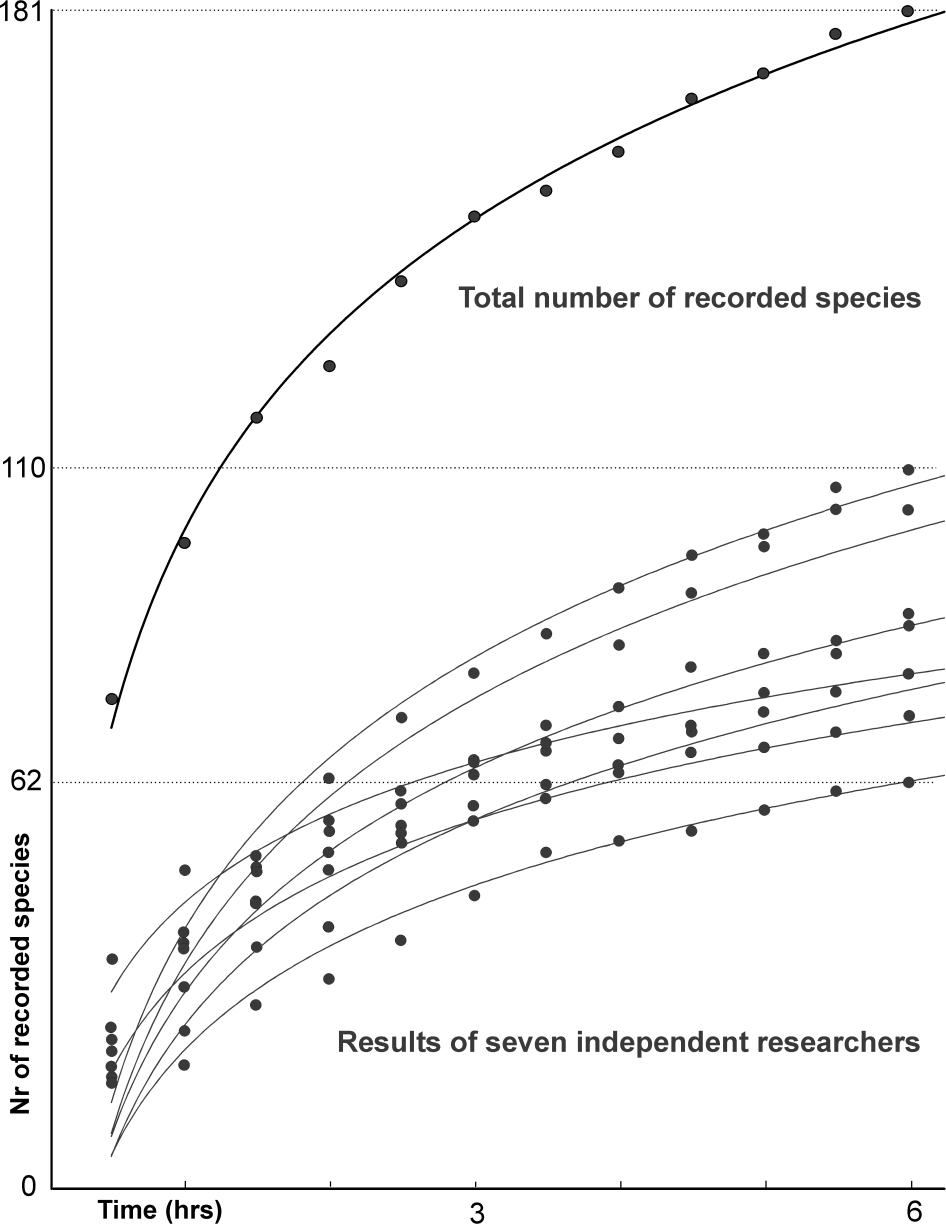
Lichen species recorded in plot 1 in twelve half-an-hour periods. Records of individual researchers (thin curves) and total records (thick) are approximated by logarithmic functions.

It has been shown that this method leads to a more complete species list, as was the case here; Table 2 and Fig 3 show the difference between records of individual researchers and all records per plot. The survey time per plot was six hours. This was not set at the outset of the study but was based on experience in plot 1. Researchers noted individual cumulative species lists in half hour periods (Fig 3), and in the 12th period on plot 1 all researchers recorded fewer than five additional species; this was taken to mean that almost all species present had been recorded and the survey of plot 1 was terminated. For subsequent plots we could have repeated this procedure of examining cumulative lists to decide the termination point, but for simplicity we decided to apply the same 6 hour survey period to each plot. The question of which approach is preferable could be investigated in some subsequent survey. Records were collated by the first three authors, who also revised and eliminated all suspicious records (possibly incorrectly identified or ambiguously identified specimens).

**Table 2:** Contrast between number of species from single researchers and the total number of recorded species.

researchers	plot 1	plot 2	plot 3	plot 4
Res. 1	110	124	134	111
Res. 2	99	106	139	90
Res. 3	85	97	136	90
Res. 4	76	61	83	65
Res. 5	78	67	96	68
Res. 6	75	82	97	75
Res. 7	62	—	94	81
Total	181	188	228	184

### Species identification and molecular barcoding

Some lichens can not be identified in the field, and field identification of some others is prone to error, so we collected specimens for almost all species; most species were collected repeatedly (S1 & S2 Tables). We identified the collected material by standard lichenological techniques (examination under the microscope, spot/UV reactions) and thin-layer chromatography (TLC) using solvent systems A, B’, C, following [42]. Our appraisals of critical specimens/species and results of TLC analyses are described in S3 Table. Specimens with ambiguous characters (morphological or chemical) and specimens that appeared to belong to undescribed species were sequenced for nrITS and/or mtSSU DNA loci. We employed the NCBI’s BLAST website [43] (http://blast.ncbi.nlm.nih.gov/Blast.cgi) to confirm their identity or at least to place them into a genus (S4 Table). Voucher specimens of all collections are deposited in the herbaria PRA (Palice and Vondrák), PRM (Bouda) and in personal herbaria of the other authors (S1 Table).

### Species and trait data for analyses

Because our concern in this study was with total biodiversity, not with how that diversity is distributed within the forest, we used only the presence or absence of lichen species in each plot (not data on abundance) to analyse the data: see S1 Table. Epiphytic and epixylic lichens, and facultatively lichenized fungi were included in the analyses. All species of the following genera are included, although some species are not lichenized: *Anisomeridium*, *Arthonia*, *Chaenothecopsis*, *Cresporhaphis*, *Cryptodiscus*, *Melaspileella*, *Mycocalicium*, *Naetrocymbe*, *Stenocybe* and *Ramonia*. We did record information on substrate, but it is not used in this analysis.

**Fig 4:**
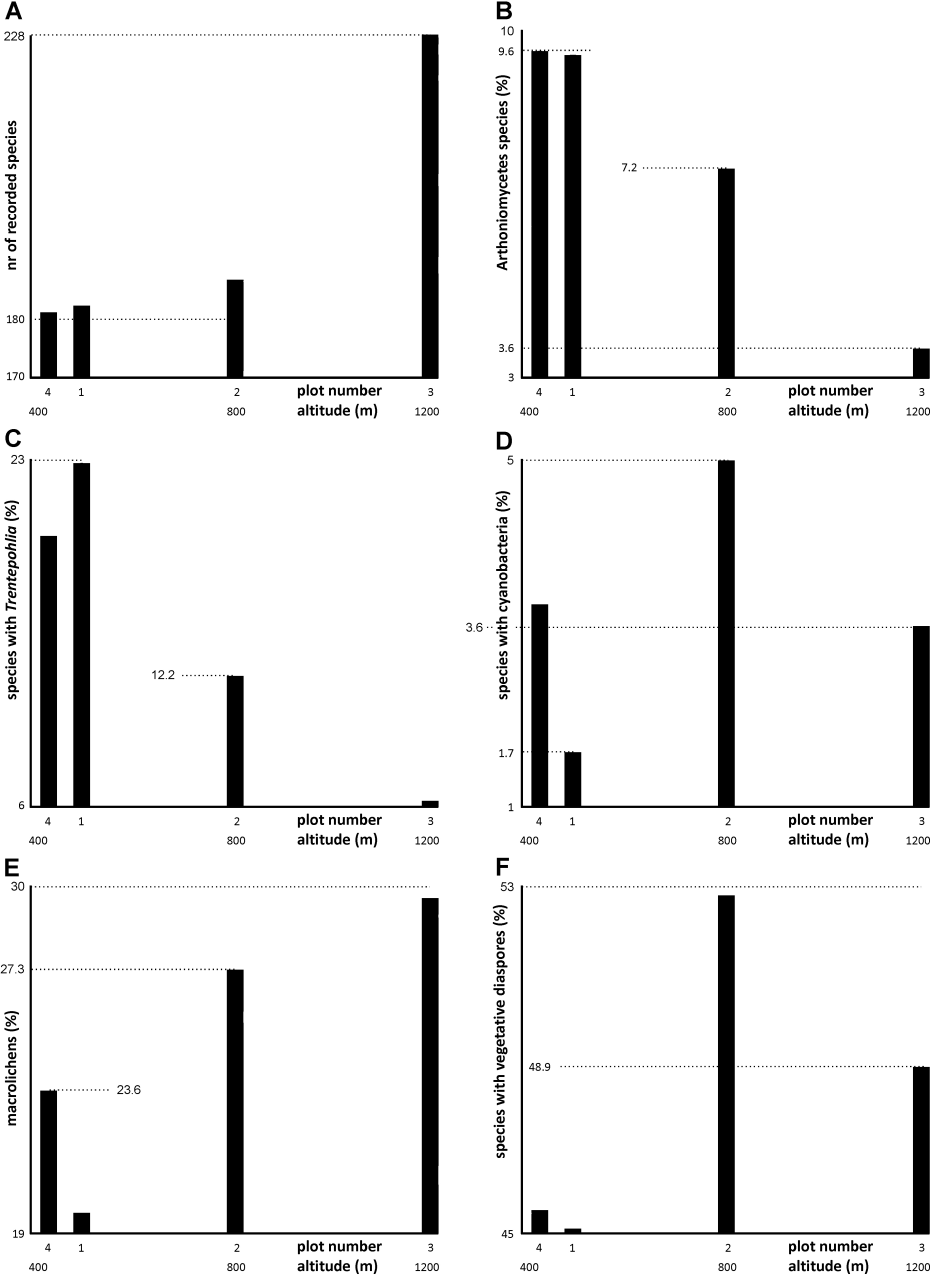
**Alpha-diversities of lichens (A, B) and the diversity within functional groups (C–F) on altitudinal gradient.** Values in charts B–F are % of all species recorded in a respective plot.

For simple analyses of functional traits (Fig 4), we employed a few basic traits commonly used in recent studies on lichen diversity [34,35,44,45]. They are: type of photobiont (cyanobacterial, trentepohlioid, others), complexity of thallus (microlichens, macrolichens) and presence/absence of vegetative diaspores.

### Data analyses

**Fig 5:**
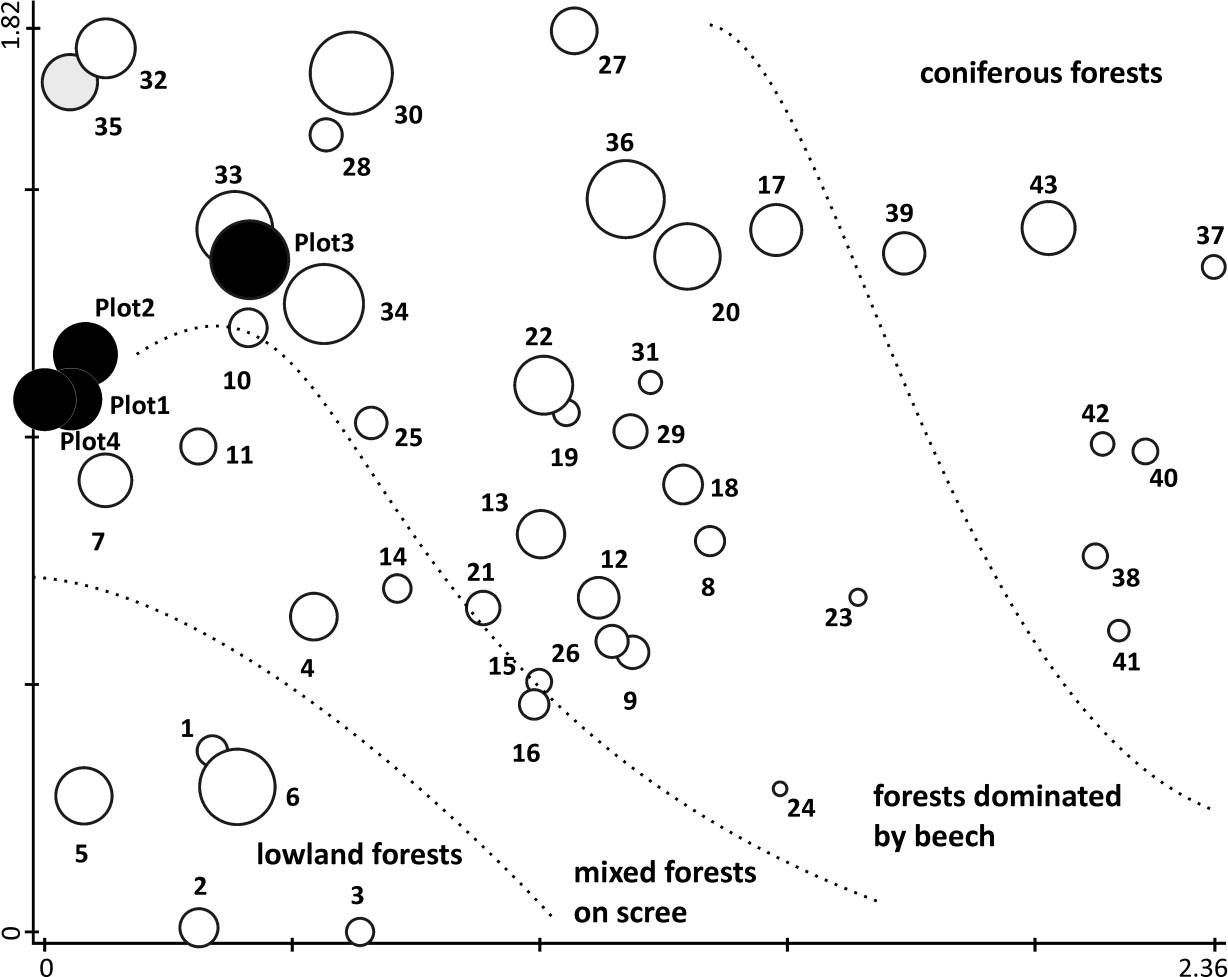
Revealed species composition compared with data available from Central European old-growth forests. DCA ordination diagram showing similarities in lichen species composition in our plots (black circles), in the previous inventory ([33]; grey circle) and in another 42 Central European old-growth forest localities (white circles). Numbers at localities correspond to S5 Table. First and second axes are plotted and explain 18.6% of the variability in species data. The size of circles corresponds to the number of species. The plot is divided by the dotted lines into four areas corresponding to the main Central European forest types.

**Fig 6:**
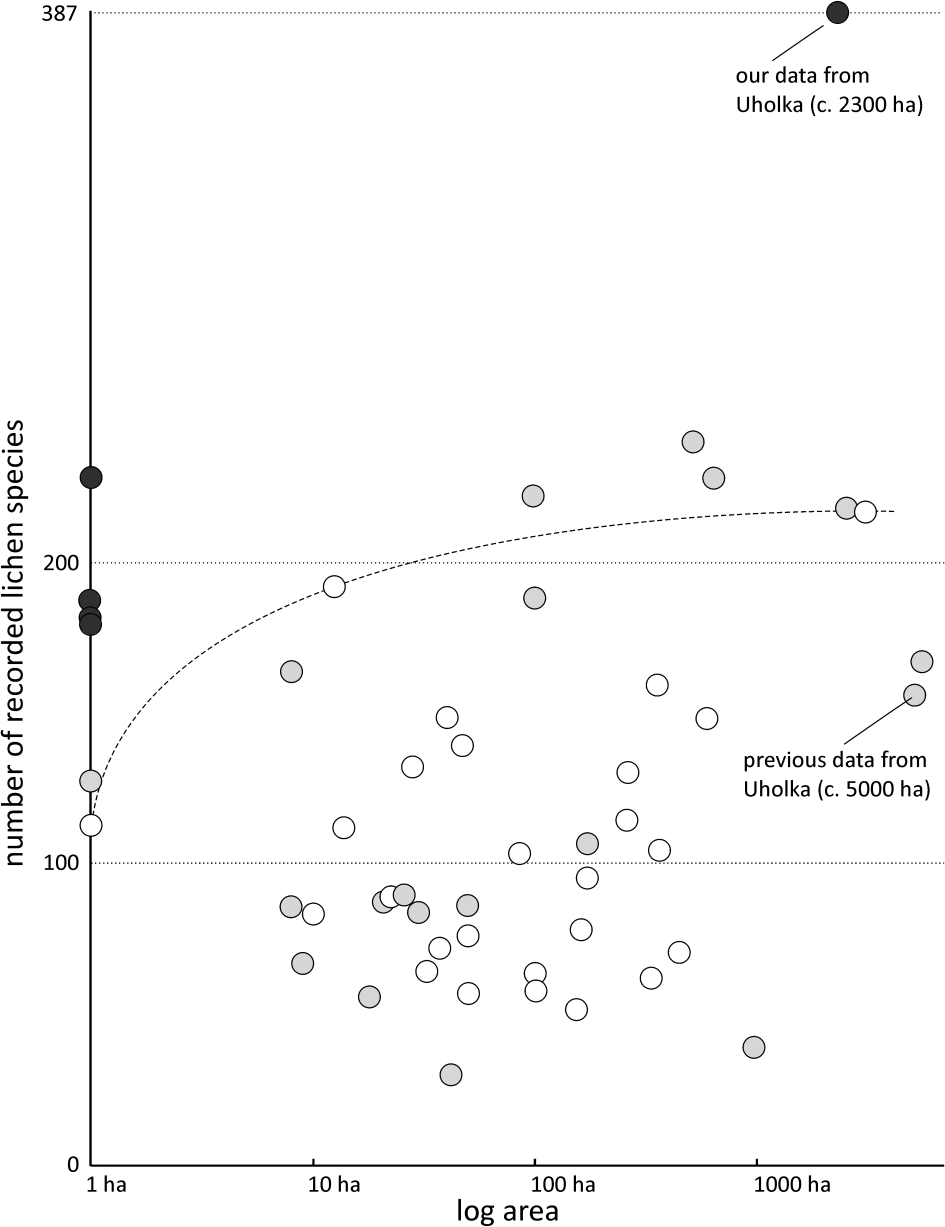
Recorded species richness in the context of data from Central European old-growth forests. Our data (black dots) showing number of species in the 1-hectare plots (alpha-diversities) and the number in the whole research area (gamma-diversity). Grey dots are data from other Central European forests dominated by beech; white dots show data from other forest types. Species/area relation for a floodplain forest surveyed by the method *multi-expert competition* [39] is drawn by the dotted line.

Our four plots were compared with each other by the number of shared lichen species and by Sørensen’s similarity index[46]. Each plot was also compared with those old-growth forest localities in Central Europe for which data is available ([15,39]; supplemented by some recent data). The whole dataset covered 43 localities and included 671 species (S5 Table). We applied the same taxonomic concepts throughout. Detrended Correspondence Analysis (DCA) in Canoco 5 [47], based on species presences/absences, was applied to display similarities (1) among our plots and (2) between our plots and other forest localities (Fig 5). Rare species were downweighted in DCA to reduce noise. In addition, species richness in our plots and in the whole studied area was compared with species richness per area obtained from 43 lichen inventories mentioned above (Fig 6).

Lists of species from each individual expert serve as incidence data usable for estimates of species richness. In other words, we may estimate how many species are present in total (i.e. including those species that were not recorded). We estimated species richness in each plot and also total species richness in the locality. We used the package Vegan for R (https://cran.r-project.org/web/packages/vegan/vegan.pdf) and employed four estimators implemented in the *specpool* function: Chao2 [48], jackknife1 & 2 [49] and bootstrap [50].

## Results

### Alpha diversities–species richness in plots

We recorded a total of 358 species in the plots. Each of our lower altitude plots (1, 2 and 4) had almost the same number of species (between 181 and 188), but plot 4, at the upper tree limit, had distinctly more species (228). Although these totals are satisfyingly high, the species richness analysis suggests that they are still far from a complete inventory (Table 3).

**Table 3:** Detected and estimated species richness. Four estimators of species richness involved in the specpool function in the R package VEGAN are employed.

dataset / incidences (data from single researchers)	detected species richness	species richness estimations for incidence data; mean/standard error			
		chao2	jackknife1	jackknife2	bootstrap
all recorded species / 7	387	442/19	450/47	484	409/28
plot 1 / 7	181	249/23	235/22	265	207/11
plot 2 / 6	188	257/21	251/28	281	220/15
plot 3 / 7	228	297/21	291/29	322	259/16
plot 4 /7	184	275/28	249/30	286	215/15

That analysis implies that we detected some 67–87% of the total number of species actually present (Table 3). The degree of completeness of our survey differs among plots. For example, plots 1 and 4 with similar numbers of detected species (181 and 184) differ in number of estimated species (249 and 275 species using Chao2 estimator). In other words, plot 1 was surveyed more effectively than plot 4 which had distinctly more rugged terrain and more varied forest structure.

### Gamma diversity–overall species richness

The total number of species recorded in all plots combined is 358. Recording outside plots yielded 251 species, but only 29 of them were not recorded within the plots (making an overall total for the forest of 387 species). The estimated number of species based on incidence data from seven researchers ranges from 409 to 484 (Table 3).

### Beta diversity–differences among local diversities in plots

Sørensen’s similarity indexes of species composition show that plot 3 (the high altitude plot) is different from all the others. Plots 1 and 4, which are close to each other and which have the same forest habitats, are the most similar (Table 4). Seventy-three species form a “common group” that occurs in all plots. This group has mostly common lichens with broad ecological amplitude, but also some rare species of old-growth forests (e.g. *Heterodermia speciosa*, *Menegazzia terebrata* and *Thelopsis rubella*). The lowland plots 1 and 4 share many species absent from other plots, e.g. the lowland species *Arthonia helvola* and *Coniocarpon cinnabarinum*. The upland plot 3 has numerous species not recorded elsewhere, some of them unexpected, including: i) the subalpine species *Caloplaca sorocarpa*, *Lecanora exspersa* and *Rinodina malangica*, ii) lichens characteristic of high montane coniferous forests, e.g. *Catillaria erysiboides*, *Frutidella pullata*, *Lecanora subintricata*, *Micarea globulosella* and iii) a few lichens that are normally saxicolous (e.g. *Acarospora fuscata*, *Circinaria caesiocinerea*, *Porpidia macrocarpa*) on bases of old beeches. Plot 2 situated on a limestone ridge has a rather heterogeneous lichen biota including sciophilous and hygrophilous as well as heliophilous xerothermic elements. The diversity of some taxonomic groups varies between plots; for example, in Arthoniomycetes diversity decreases strongly with increasing altitude (Fig 4B).

**Table 4:** Number of shared species (above diagonal) and Sørensen’s similarity indexes (below diagonal) for all pairs of plots.

	plot 1	plot 2	plot 3	plot 4
plot 1	–	119	102	136
plot 2	0.65	–	132	113
plot 3	0.5	0.63	–	102
plot 4	0.75	0.62	0.5	–

The proportion of lichens with trentepohlioid photobiont decreases considerably with increasing altitude (Fig 4C). The proportion of lichens with cyanobacterial photobiont is always low, though slightly raised in plot 2, which is influenced by its limestone bedrock and which has trees with slightly acidic to subneutral bark pH, e.g. *Acer platanoides* (Fig 4D). Macrolichens (i.e. foliose and fruticose lichens) are infrequent in all plots (c. 20–30%), but their proportion increases with altitude (Fig 4E), as they prefer higher humidity [34]. The proportion of species with vegetative diaspores is rougly constant, about 50%, in all plots (Fig 4F).

### Uholka in the context of Central European forests

Lichen species composition in plots 1, 2 and 4 is most similar to deciduous mixed forests on limestone in the Muránska Planina Mts in the Western Carpathians in Slovakia (locs 7 and 11 in Fig 5; [51,52]), but the higher altitude plot 3 is more similar to the Eastern Carpathian beech forest Stužica / Stuzhitsa (locs 33 and 34; [15]) and to an upland mixed forest in Hrdzavá dolina in Slovakia (loc. 10; [53]).

## Discussion

### Advantages and disadvantages of the new method

The trial at Uholka used a method which consists of exhaustive, multi-expert competitive sampling of 1-hectare moveable local hot-spots. It is an effective, practical method for obtaining representative datasets in biodiversity research. It can be applied in (almost) any type of forest in (almost) any region. The substantial increase in the number of species found by our method compared to the prior survey of the same area (357 versus 161) strongly suggests that our method works better than other survey methods that lichenologists have used. In contrast with conventional methods, our method has the following advantages:

1. **Movable character of the hot spot plots**. What we are calling a hot spot is basically any area that is very different from the bulk of the forest in ways that support species richness. Such areas are not fixed in position. They may—indeed, they almost certainly will—change over time, influenced by factors including natural disturbances, the presence of dying and overaged trees, accumulations of dead wood, etc. For that reason, a future survey of the same forest need not use the same plots that we used. If a substantial period of time has elapsed, then almost certainly it *should not* use the same plots. (This is why we use the expression *movable* hot-spot plots.)
2. Replicability. Theoretically, it is possible that selection of different hot spots in some future survey could result in a very different inventory of species than ours. In reality, we would not expect this to happen. First, we expect that similar hotspots, based on our 6 criteria, will give similar species. (We assume, of course, that future surveyors will make a sensible selection of hotspots, but this is not a particularly demanding task and does not require an unreasonably high level of skill.) Second, we believe our method has the potential to provide even greater replicability than conventional methods, as a simple consequence of the fact that our method samples the common species at least as well as conventional methods, but it samples the rare species much more thoroughly.
3. It yields more complete lists of species. 4) Data from a few hot-spots captures almost all of the diversity present within the entire forest. It will include most of those species that are widely distributed in the forest, either because their preferred substrate/habitat is widely distributed or because they are indifferent as to substrate/habitat. It will include most of the species that have specialised substrate/habitat requirements but which are fairly common when those requirements are met, because we have deliberately sought out these specialised substrates/habitats. However, it will include only some of the species that have specialised requirements but which are rare even when those requirements are met. (These are typically species that have only a low population in the forest. They may occur at only very few sites, and whether or not they are recorded in any survey, even a focussed one like ours, is largely a matter of chance.) A conventional survey may do as well as ours for the first group of species, but it will do less well on the second and third groups. We expect that a repeated survey using our method at some future date will obtain an inventory that is similar to ours for the first two groups of species, but it may differ considerably for the third group, because the results for that group are more strongly influenced by chance factors. Species withIt will also include 5) Data from any hot-spot is directly comparable with data from other hot-spots. 6) Data from hot-spots can be compared in a useful way with data from larger plots/sites (even though the two datasets are not 100% compatible). 7) The data can be used to make statistical estimates of the total number of species present (i.e. including those that the survey failed to record). 8) The gaps in knowledge (or other kinds of "blind spot") of an individual lichenologist do not bias the results, because other recorders in the team compensate for them. 9) Each member of the team is likely to have specialised knowledge that the others lack, and may be able to record rare, or substrate-specific, or inconspicuous species, or species in difficult taxonomic groups that the other recorders would overlook. 10) Working in a team allows more expertise to be brought to bear on the identification of difficult specimens. 11) Field research is time-efficient. Only one (for a small forest) or a few (for a large forest) plots are required to survey a forest.

There are some limitations:

- The method requires a team of experienced experts. b) All doubtful determinations made by the original team must be followed up by careful revision of the collections concerned, which can be time consuming. c) The small number of plots employed means that some kinds of statistical analyses can not be applied. d) Comparisons made among such plots must consider the possibility that selection of different hot-spots may yield slightly different results. (This could be tested, by replicating the study locally, though the effort involved might be excessive.)

### Our lichen diversity survey vs. previous research in the locality

Grid ecological sampling (Fig 1) of lichen biota in the entire Uholka forest [33] resulted in a list of 161 lichen species. Their total sampling area was about eight hectares (163 plots × 500 m^2^; see Table 5). Although they studied twice the area that we did, they recorded only about 45% as many species (Fig 6). Their survey differs considerably in species composition within some genera, e.g. *Arthonia*, *Biatora*, *Caloplaca* and *Micarea* (Table 5). They did record 24 species that we did not (though at least one third of that figure probably arises from different determinations of the same species). As noted above, we are well aware that the species list we obtained, though extensive, is not complete. Floristic differences are thoroughly discussed in Malíček et al. [40].

**Table 5:** Comparison between our survey of lichen diversity and the previous research in the same area (Uholka in Fig 1).

parameters	Previous survey ([33]; only data from the part "Uholka"; see Fig 1)	Our data
**Field research**		
nr of plots	163	4
size / shape of plots	500 m2 / round plots with diam. c. 25 m	10.000 m2 / square plots
method of plot design	non-stratified systematic cluster sampling (Fig 1)	aimed to local habitat diversity hot-spots & to maximize beta-diversity
total area of plots / area of study	8.15 ha / c. 5000 ha	4 ha / c. 2300 ha
**Detected lichen species richness**		
nr of recorded lichen species	156	358 (in four 1 ha plots) / 387 (with records outside plots)
nr of species per plot: min—mean—max	1 - <20–40	181–195–228
portion of macrolichens in the species richness dataset	41% (64 of 156)	24% (91 of 381)
Nr of *Arthonia* species	3	13
Nr of *Biatora* species	5	13
Nr of *Caloplaca* (s.lat.) species	2	12
Nr of *Micarea* species	0	19

The percentage of macrolichens in beech-dominated forests is estimated to be about 27% [15]. It is 24% in our dataset from Uholka, but 41% in the previous survey. The high portion of macrolichens in the previous survey, together with the obvious imperfect detection within some microlichen genera (Table 5), suggest that a conventional survey tends to be a somewhat superficial survey.

### Underestimated species richness in Central European old-growth forests

We do not claim that the Uholka forest has a distinctly higher lichen diversity than any other Central European forest, although a naive interpretation of our results might suggest that conclusion (Fig 6). For example 228 species in plot 3 in Uholka is comparable with the highest numbers from large beech forest areas: 228 species per 630 ha [15] and 222 species per 102 ha [27]. We do claim that our survey method is superior to others and that this is a sufficient explanation for the differences. We suspect that the more humid Shyrokyi Luh forest (Fig 1), where Dymytrova et al. [33] recorded more species than in Uholka, is more species rich, while slightly smaller old-growth forest remnants, such as the Slovakian Stužica [15], Ukrainian Stuzhitsa [54–56] and Austrian Rothwald [57–59] may have comparable species richness per area. Forests dominated by beech (grey dots in Fig 6) are obviously more species rich than other forest types (white dots in Fig 6) which corresponds with results by Hofmeister et al. [4] considering old beech trees as a “lifeboat” for lichen diversity in Central European forests. However, some lowland forests are also known to be species rich (see the dotted line in Fig 6, which refers to a floodplain forest in the Czech Republic; [39]). These data were obtained by the multi-expert competitive survey, but without employing the search for diversity hot-spot plots.

## Conclusions

We improved methods for recording epiphytic lichen diversity in forests so as to maximise the number of species detected per fixed area. Our method involves subjective selection of 1-hectare plots in local diversity hot-spots and a multi-expert competitive approach. It produces mutually comparable data and it appears to be substantially more efficient for assessment of species richness than methods used previously for ecological sampling or taxonomic surveys.

A detailed survey in the largest primeval forests in Central Europe, “Uholka-Shyrokyi Luh”, revealed unexpectedly high lichen diversity: 228 species recorded from a single 1-hectare plot is equal to the highest number of species recorded in Central European forests of far larger sizes. Gamma diversity revealed in the studied area (387 species) greatly exceeds all previous data from European forest localities. It also exceeds by more than a factor of two the Gamma diversity recorded from the same area by the previous detailed inventory employing a systematic grid sampling. We wish to stress the importance of local diversity hot-spots for lichen inventories and that such spots are usually sparse and unevenly distributed within a locality and must be deliberately searched for. In our opinion, any survey that does not pay particular attention to hot-spots will substantially under-estimate the number of species present. A future goal is a detailed evaluation of differences among species lists from different hot spots in the same locality.

## Supporting information

S1 Table Diversity data from the research in plots (1‒4) and outside plots (out). Substrate abbreviations correspond with the Table 1. Vouchers are indicated by initials of the authors. Vouchers with asterisk are with TLC data; those with exclamation mark were sequenced (see S3 and S4 Tables). The nomenclature follows Hafellner & Türk (2016) [The lichenized fungi of Austria] and Wirth et al. (2013) [Die Flechten Deutschlands] in case of taxa missing in the former study. Species absent from both publications are provided by author initials.
(XLS)

S2 Table Species lists made by individual researchers in plots 1‒4. (XLSX)

S3 Table Notes to identifications and TLC results. (XLSX)

S4 Table Identifications of specimens according to NCBI's Blast results. NCBI's accession numbers are attached.
(XLSX)

S5 Table The summary of lichen inventories in Central European old-growth forests, employed in Figs 5 and 6. Localities are sorted according to forest types. Five groups of forest types are separated by horizontal lines; from above: lowland forests, maple-lime scree forests, beech-dominated forests and coniferous forests. Tree species abbreviations correspond with the Table 1. See the list of references below the table.
(XLSX)

## References

[1] Loo JA. The role of forests in the preservation of biodiversity. In: Forests and forest plants. Vol. 3 (ed. by Owens J. and Lund H.). EOLSS Publishers Co Ltd; 2009.

[2] Boch S, Prati D, Hessenmöller D, Schulze ED, Fischer M. Richness of lichen species, especially of threatened ones, is promoted by management methods furthering stand continuity. PLOS One 2013; 8: e55461 10.1371/journal.pone.0055461 23383196 PMC3558497

[3] Hofmeister J, Hošek J, Brabec M, Dvořák D, Beran M, Deckerová H, et al Value of old forest attributes related to cryptogam species richness in temperate forests: A quantitative assessment. Ecol Indic 2015; 57: 497–504.

[4] Hofmeister J, Hošek J, Malíček J, Palice Z, Syrovátková L, Steinová J, et al Large beech (Fagus sylvatica) trees as ‘lifeboats’ for lichen diversity in central European forests. Biodivers Conserv 2016; 25: 1073–1090.

[5] Kubiak D, Osyczka P, Rola K. Spontaneous restoration of epiphytic lichen biota in managed forests planted on habitats typical for temperate deciduous forest. Biodivers Conserv 2016; 25: 1937–1954.

[6] Kaufmann S, Hauck M, Leuschner C Comparing the plant diversity of paired beech primeval and production forests: Management reduces cryptogam, but not vascular plant species richness. Forest Ecol Manage 2017; 400: 58–67.

[7] Johansson P, Gustafsson L Red-listed and indicator lichens in woodland key habitats and production forests in Sweden. Can J For Res 2001; 31: 1617–1628.

[8] Paillet Y, Bergès L, Hjältén J, Odor P, Avon C, Bernhardt-Römermann M, et al Biodiversity Differences between Managed and Unmanaged Forests: Meta-Analysis of Species Richness in Europe. Conserv Biol 2010; 24: 101–112. 10.1111/j.1523-1739.2009.01399.x 20121845

[9] Tibell L Crustose lichens as indicators of forest continuity in boreal coniferous forests. Nord J Bot 1992; 12: 427–450.

[10] Selva SB Using calicioid lichens and fungi to assess ecological continuity in the Acadian Forest Ecoregion of the Canadian Maritimes. Forestry Chron 2003; 79: 550–558.

[11] Peterson EB, McCune B The importance of hot-spots for lichen diversity in forests of western Oregon. Bryologist 2003; 106: 246–256.

[12] Newmaster SG, Belland RJ, Arsenault A, Vitt DH, Stephens TR The ones we left behind: Comparing plot sampling and floristic habitat sampling for estimating biodiversity. Divers Distrib 2005; 11: 57–72.

[13] Dymytrova L, Nadyeina O, Hobi ML, Scheidegger C Topographic and forest-stand variables determining epiphytic lichen diversity in the primeval beech forest in the Ukrainian Carpathians. Biodivers Conserv 2014; 23: 1367–1394.

[14] Neitlich PN, McCune B Hot-spots of Epiphytic Lichen Diversity in Two Young Managed Forests. Conserv Biol 1997; 11: 172–182.

[15] Vondrák J, Malíček J, Šoun J, Pouska V Epiphytic lichens of Stužica (E Slovakia) in the context of Central European old-growth forests. Herzogia 2015; 28: 104–126.

[16] Kuusinen M, Siitonen J Epiphytic lichen diversity in old-growth and managed Picea abies stands in southern Finland. J Veg Sci 1998; 9: 283–292.

[17] Fritz Ö, Heilmann-Clausen J Rot holes create key microhabitats for epiphytic lichens and bryophytes on beech (Fagus sylvatica). Biol Conserv 2010; 143: 1008–1016.

[18] Larrieu L, Paillet Y, Winter S, Bütler R, Kraus D, Krumm F, et al Tree related microhabitats in temperate and Mediterranean European forests: A hierarchical typology for inventory standardization. Ecol Indic 2018; 84: 194–207.

[19] Ellis CJ Lichen epiphyte diversity: A species, community and trait-based review.—Perspect Plant Ecol Evol Syst 2012; 14: 131–152.

[20] Nascimbene J, Dainese M, Sitzia T Contrasting responses of epiphytic and dead wood-dwelling lichen diversity to forest management abandonment in silver fir mature woodlands. For Ecol Manage 2013; 289: 325–332.

[21] Ódor P, Király I, Tinya F, Bortignon F, Nascimbene J Patterns and drivers of species composition of epiphytic bryophytes and lichens in managed temperate forests. For Ecol Manage 2013; 306: 256–265.

[22] Horak J, Vodka S, Kout J, Halda JP, Bogusch P, Pech P Biodiversity of most dead wood-dependent organisms in thermophilic temperate oak woodlands thrives on diversity of open landscape structures. Forest Ecol Manage 2014; 315: 80–85.

[23] Boch S, Müller J, Prati D, Blaser S, Fischer M Up in the tree–The overlooked richness of bryophytes and lichens in tree crowns. PLOS One 2013a; 8: e84913. 24358373 10.1371/journal.pone.0084913 PMC3866205

[24] Kiebacher T, Keller C, Scheidegger C, Bergamini A Hidden crown jewels: the role of tree crowns for bryophyte and lichen species richness in sycamore maple wooded pastures. Biodivers Conserv 2016; 25: 1605–1624.

[25] Ellis CJ, Coppins BJ Taxonomic survey compared to ecological sampling: are the results consistent for woodland epiphytes?. Lichenologist 2017; 49: 141–155.

[26] Guttová A, Palice Z, Czarnota P, Halda JP, Lukáč M, Malíček J, et al Lišajníky Národného parku Muránska planina IV–Fabova hoľa. [Lichens of the Muránska Planina National Park IV–Fabova hoľa]. Acta Rer Nat Mus Nat Slov 2012; 43: 51–76.

[27] Malíček J, Palice Z Lichens of the virgin forest reserve Žofínský prales (Czech Republic) and surrounding woodlands. Herzogia 2013; 26: 253–292.

[28] Giordani P, Brunialti G, Benesperi R, Rizzi G, Frati L, Modenesi P Rapid biodiversity assessment in lichen diversity surveys: implications for quality assurance. J Environ Monit 2009; 11: 730–735. 10.1039/b818173j 19557222

[29] Moning C, Werth S, Dziock F, Bassler C, Bradtka J, Hothorn T, et al Lichen diversity in temperate montane forests is influenced by forest structure more than climate. For Ecol Manage 2009; 258: 745–751.

[30] Svoboda D, Peksa O, Veselá J Epiphytic lichen diversity in central European oak forests: Assessment of the effects of natural environmental factors and human influences. Environ Pollut 2010; 158: 812–819. 10.1016/j.envpol.2009.10.001 19880227

[31] Nascimbene J, Marini L, Nimis PL Epiphytic lichen diversity in old-growth and managed Picea abies stands in Alpine spruce forests. For Ecol Manage 2010; 260: 603–609.

[32] Nascimbene J, Nimis PL, Dainese M Epiphytic lichen conservation in the Italian Alps: the role of forest type. Fungal Ecol 2014; 11: 164–172.

[33] Dymytrova L, Nadyeina O, Naumovych A, Keller C, Scheidegger C Primeval beech forests of Ukrainian Carpathians are sanctuaries for rare and endangered epiphytic lichens. Herzogia 2013; 26: 73–89.

[34] Bässler C, Cadotte MW, Beudert B et al Contrasting patterns of lichen functional diversity and species richness across an elevation gradient. Ecography 2016; 39: 689–698.

[35] Benítez A, Aragón G, González Y, Prieto M Functional traits of epiphytic lichens in response to forest disturbance and as predictors of total richness and diversity. Ecol Indic 2018; 86: 18–26.

[36] Hunter ML Jr, Webb SL Enlisting taxonomists to survey poorly known taxa for biodiversity conservation: a lichen case study. Conserv Biol 2002; 16: 660–665.

[37] Hafellner J, Komposch H Diversität epiphytischer Flechten und lichenicoler Pilze in einem mitteleuropäischen Urwaldrest und einem angrenzenden Forst. Herzogia 2007; 20: 87–113.

[38] Lõhmus P, Leppik E, Motiejūnaite J, Suija A, Lõhmus A Old selectively cut forests can host rich lichen communities–lessons from an exhaustive field survey. Nova Hedwig 2012; 95: 493–515.

[39] Vondrák J, Malíček J, Palice Z, Coppins BJ, Kukwa M, Czarnota P, et al Methods for obtaining more complete species lists in surveys of lichen biodiversity. Nord J Bot 2016; 34: 619–626.

[40] Malíček J, Palice Z, Acton A, Berger F, Bouda F, Sanderson N, et al Uholka primeval forest in the Ukrainian Carpathians–a keynote area for diversity of forest lichens in Europe. Herzogia. Forthcoming 2018.

[41] Commarmot B, Brändli U-B, Hamor F, Lavnyy V Inventory of the largest primeval beech forest in Europe–a Swiss-Ukrainian scientific adventure. WSL Swiss Federal Research Institute, Birmensdorf, Switzerland; 2013.

[42] Orange A, James PW, White FJ Microchemical methods for the identification of lichens. British Lichen Society, London; 2010.

[43] Johnson M, Zaretskaya I, Raytselis R, Merezhuk Y, McGinnis S, Madden TL NCBI BLAST: a better web interface. Nucl Acids Res 2008; 36 (suppl 2): W5–W9. 18440982 10.1093/nar/gkn201 PMC2447716

[44] Nelson PR, McCune B, Roland C, Stehn S Non-parametric methods reveal non-linear functional trait variation of lichens along environmental and fire age gradients. J Veg Sci 2015; 26: 848–865.

[45] Wolseley P, Sanderson N, Thüs H, Carpenter D, Eggleton P Patterns and drivers of lichen species composition in a NW-European lowland deciduous woodland complex. Biodivers Conserv 2017; 26: 401–419.

[46] Sørensen T A method of establishing groups of equal amplitude in plant sociology based on similarity of species content. Biologiska Skrifter 1948; 5: 1–34.

[47] ter Braak CJF, Šmilauer P Canoco reference manual and user's guide: software for ordination, version 5.0. Microcomputer Power, Ithaca; 2012.

[48] Chao A Estimating the population size for capture-recapture data with unequal catchability. Biometrics 1987; 43: 783–791. 3427163

[49] Burnham KP, Overton WS Estimation of the size of a closed population when capture probabilities vary among animals. Biometrika 1978; 65: 625–633.

[50] Chao A, Gotelli NJ, Hsieh TC, Sander EL, Ma KH, Colwell RK, et al Rarefaction and extrapolation with Hill numbers: a framework for sampling and estimation in species diversity studies. Ecol Monog 2014; 84: 45–67.

[51] Guttová A, Palice Z Lišajníky Národného parku Muránska planina II–Javorníková dolina. [Lichens of National Park Muránska planina II—the Javorníková dolina Valley]. Výskum a ochrana prírody Muránskej Planiny 2002; 3: 53–68.

[52] Guttová A, Palice Z Lišajníky Národného parku Muránska planina III–Cigánka. [Lichens of the Muránska Planina National Park III–Cigánka]. Reussia 2005; 1 (Suppl. 1, 2004): 11–47.

[53] Guttová A, Palice Z Lišajníky Národného parku Muránska planina I–Hrdzavá dolina [Lichens of National Park Muránska planina I—the Hrdzavá dolina Valley]. Výskum a Ochrana Prírody Muránskej Planiny 1999; 2: 35–47.

[54] Kondratyuk SY, Coppins BJ Basement for the lichen monitoring in Uzhansky national nature park, Ukrainian part of the Biosphere reserve "Eastern Carpathians". Rocz Bieszczad 2000; 8 (1999): 149–191.

[55] Kondratyuk SY, Coppins BJ, Zelenko SD, Khodosovtsev AY, Coppins AM, Wolseley PA Lobarion lichens as indicators of primeval forests in the Ukrainian part of the International Biosphere Reserve “Eastern Carpathians”: distribution, ecology, long-term monitoring and recommendations for conservation. Rocz Bieszczad 1998; 6 (1997): 65–87.

[56] Motiejūnaitė J, Zalewska A, Kukwa M, Fałtynowicz W New for Ukraine or interesting lichens and allied fungi from the Regional Landscape Park "Stuzhytzia". Ukr Bot J 1999; 56: 596–600.

[57] Bilovitz PO Zur Flechtendiversität des "Mariazellerlandes" und ausgewählter Standorte im Bereich Naßköhn-Hinteralm (Nordalpen, Steiermark). Mitt Naturwiss Ver Steiermark 2007; 136: 61–112.

[58] Türk R, Breuss O Flechten aus Niederösterreich I. Steirisch-niederösterreichische Kalkalpen. Verh Zool-Bot Ges Österr 1994; 131: 79–96.

[59] Türk R Flechten im Wildnisgebiet Dürrenstein. Silva Fera 2015; 4: 26–40.

